# Prescribing Intensity in Resistance Training Using Rating of Perceived Effort: A Randomized Controlled Trial

**DOI:** 10.3389/fphys.2022.891385

**Published:** 2022-04-29

**Authors:** Yael Boxman-Zeevi, Hadar Schwartz, Itai Har-Nir, Nadia Bordo, Israel Halperin

**Affiliations:** ^1^ Department of Health Promotion, Sackler Faculty of Medicine, Tel-Aviv University, Tel-Aviv, Israel; ^2^ Sylvan Adams Sports Institute, Tel Aviv University, Tel-Aviv, Israel; ^3^ Faculty of Industrial Engineering and Management, Technion—Israel Institute of Technology, Haifa, Israel

**Keywords:** adherence, autonomy, intensity-prescription, effort-regulation, online exercise

## Abstract

**Introduction:** Rating of perceived effort (RPE) scales are used to prescribe intensity in resistance training (RT) in several ways. For instance, trainees can reach a specific RPE value by modifying the number of repetitions, lifted loads, or other training variables. Given the multiple approaches of prescribing intensity using RPE and its growing popularity, we compared the effects of two RPE prescription approaches on adherence rates, body composition, performance and psychological outcomes, in an online RT intervention.

**Methods:** We randomly assigned 57 healthy participants without RT experience (60% females, age range: 18–45) to one of two groups that received two weekly RT sessions using a resistance-band for 8 weeks. In the fixed-repetition group, participants adjusted the band resistance with the goal of completing 10 repetitions and reaching a 7-RPE on a 0–10 scale by the 10th repetition. In the open-repetition group, participants selected their preferred band resistance and completed repetitions until reaching a 7-RPE by the final repetition. We measured body composition, performance, and program satisfaction rates.

**Results:** We assessed 46 participants at post-test, 24 from the fixed-repetition group and 22 from the open-repetition group. We observed non-significant and trivial differences between groups in all outcomes (*p* > 0.05). We then combined the pre-post change scores of the two groups. We found that adherence rates began at 89% and gradually decreased to 42%. On average, participants increased their fat-free mass [0.3 kg (95% CI: 0.1–0.6)], isometric mid-thigh pull [5.5 kg (95% CI: 0.8–10.4)], isometric knee-extension [2.2 kg (95% CI: 0.8–3.7)], and push-ups [6.3 repetitions (95% CI: 4.5–8.2)]. We observed non-significant and trivial changes in bodyweight, grip-force, and countermovement jump. Participants reported high satisfaction rates with all components of the program.

**Conclusion:** Participants in both groups improved their body composition and physical capacity to a similar extent, and reported comparable satisfaction rates with the programs they followed. Accordingly, either prescription approach can be used to deliver online RT sessions based on personal preferences and logistical constraints. However, since adherences rates declined over the course of the study, future research should test additional strategies aiming to maintain adherence rates.

**Clinical Trial registration:** https://clinicaltrials.gov/ct2/show/NCT04895865?term=NCT04895865&draw=2&rank=1, identifier NCT04895865

## 1 Introduction

Resistance training (RT) has many health benefits, such as reducing the rates of different diseases and all-cause mortality (Maestroni et al., 2020; Kraschnewski et al., 2016). While health organizations recommend two weekly sessions of RT (American College of Sports Medicine, 2009; American College of Sports Medicine et al., 2018), only 4 to 30 percent of the population follow these guidelines (Bennie et al., 2018; Harada et al., 2008). A possible explanation for the low RT participation rates is the generic nature of the standard RT prescription approach (Shimano et al., 2006; Richens and Cleather, 2014; Phillips and Winett, 2010). Specifically, the resistance that trainees are expected to use is commonly fixed and calculated as a percentage of the maximal load that can be lifted once, (i.e., one Repetition Maximum [1RM])^1^ (American College of Sports Medicine et al., 2009; American College of Sports Medicine et al., 2018). The number of repetitions prescribed per set is also commonly fixed and selected from a narrow range (American College of Sports Medicine et al., 2009; American College of Sports Medicine et al., 2018). For example, novice trainees aiming to gain muscular strength, mass, and endurance, are recommended to use resistance equivalent to 60%–80% of their 1RM and perform 8–12 repetitions per set (American College of Sports Medicine et al., 2009; American College of Sports Medicine et al., 2018).

Following these restrictive guidelines can lead to inconsistencies in the difficulty level among trainees who follow the same program. This is because the maximum number of repetitions that trainees can complete differs considerably, even when using the same relative resistance (e.g., 70% 1RM) (Shimano et al., 2006; Richens and Cleather, 2014). Furthermore, some trainees prefer to participate in short, high-intensity workouts rather than long, low-intensity ones, whereas others prefer the opposite (Teixeira et al., 2021; Ekkekakis et al., 2005). While considering trainee preferences has a range of benefits (Halperin et al., 2018; Jaitner and Mess, 2019), the standard approach to RT does not account for trainee preferences regarding load and repetition range. Collectively, these characteristics of the standard approach may partly contribute to the low RT participation and adherence rates.

An alternative to the standard approach is to prescribe RT using single-item rating of perceived effort (RPE) scales that typically range from 0 (“no effort”) to 10 (“maximum effort”) (Buckley and Borg, 2011; Schwartz et al., 2021b). RPE is commonly used in RT with the fixed-repetition approach; the trainer or trainee selects the external resistance that is expected to produce a certain RPE value after completing a predetermined number of repetitions (Tiggemann et al., 2016; Stojanović et al., 2021). For example, reaching an RPE of 7/10 by the final, 10th repetition. If the RPE reached is lower or higher than the target 7/10 by the end of the set, trainees adjust the resistance for the subsequent sets (i.e., load when using weights and tension when using resistance bands). Under the fixed-repetition approach, trainees know exactly how many repetitions they are required to complete, which has been shown to improve performance compared to less certain set endpoints (Halperin et al., 2014; Billaut et al., 2011). However, trainees are required to anticipate what their RPE will be by the end of the set before it begins—a task that may be difficult to execute with sufficient accuracy (Halperin et al., 2021).

Another alternative is the open-repetition approach, in which trainees select the resistance and terminate a set once they have reached a certain RPE value, irrespective of how many repetitions were required to reach that RPE value (Schwartz et al., 2021b). For example, assume that two trainees are using the same relative external resistance, and are guided to terminate a set when reaching an RPE of 7/10. For one trainee this may happen after 8 repetitions and for the other it may be after 12. Importantly, one can reach the same RPE by using different blends of resistance and repetitions (e.g., high resistance coupled with fewer repetitions and vice versa). By allowing trainees to self-select the resistance, the open-repetition approach better accounts for their load preferences. Provided that sufficient effort is invested in each set, the exact blend of resistance and repetition is less important for gaining benefits from RT (Csapo and Alegre, 2016; Mitchell et al., 2012; Morton et al., 2016). Under the open-repetition approach, trainees monitor their effort during the set, so they are not required to anticipate what their RPE will be before set initiation. However, trainees do not have a clear set endpoint. Both RPE approaches account for individual abilities by having trainees adjust certain RT variables, but they do so in different ways, which may lead to different outcomes. No study to date has compared the effects of the two RPE approaches on adherence rates, body composition, performance and psychological outcomes.

Alongside the rigidness associated with the standard RT prescription approaches, other factors are negatively associated with low participation rates: lack of time, limited instructions on exercise execution and progression, and shortage of equipment and facilities (Rhodes et al., 2017). These factors can be addressed by delivering live, online, home-based RT sessions using resistance bands. Despite its potential, relatively few studies have implemented and examined this approach of delivering RT sessions (Aksay, 2021; Ibrahim et al., 2021; Mascarenhas et al., 2018; Carlson et al., 2022). Accordingly, the primary aim of this study was to compare the fixed and open repetition RPE approaches, delivered live and online using resistance bands in a sample of healthy adults, on adherence, body composition, performance, and psychological outcomes. The secondary aim of this study was to examine the effect of the RT sessions across groups on the same outcomes. We did not have a prior hypothesis as to which approach would be better; however, we did hypothesize that all outcomes would improve following the intervention.

## 2 Materials and Methods

### 2.1 Experimental Design

A parallel arm randomized controlled trial was implemented and conducted in Israel. The study was approved by the Ethics Committee of Tel-Aviv University (approval number: 0002205-1) and the trial was pre-registered at ClinicalTrials.gov (NCT04895865).

### 2.2 Participants

**FIGURE 1:**
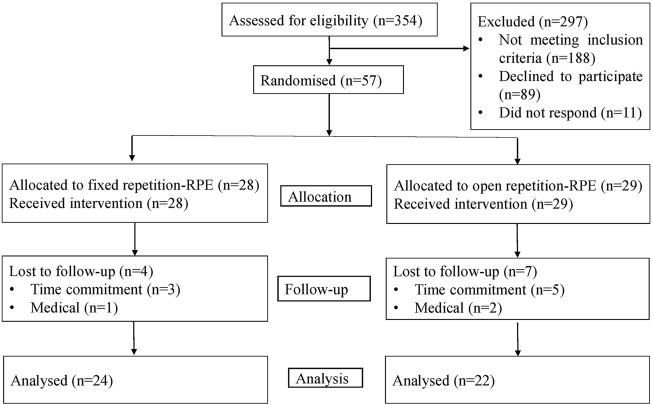
Participant flowchart.

We aimed to recruit a total of 60 participants (30 per group) based on our resources (Lakens, 2022), and previous experience with this type of intervention (Schwartz et al., 2021b). We were limited in our ability to test more than 60 participants in a two-week period in the pre- and post-tests, and expected that 30 per group would allow the instructor to oversee all participants. Eligible participants included healthy, sedentary, adults (ages: 18–45) with no RT experience. Exclusion criteria were any co-morbidities preventing participation in the program, routine use of prescription medication, pregnancy or delivery within the past 6 months. Participants were recruited through Facebook and Instagram (Meta, Ca, United States) during April and May, 2021. Eligible participants were provided with general information about the study and underwent a health screening using the Physical Activity Readiness Questionnaire (Warburton et al., 2011) which was translated to Hebrew by the Israel Ministry of Health. Eventually, 57 participants were block-randomized using RAND function in excel (Microsoft Corporation, WA, United States) based on their age and sex to one of two groups: fixed-repetition RPE (10 males and 18 females, age: 35 ± 7 years, weight: 71 ± 16 kg, height: 168 ± 7 cm) and open-repetition RPE (12 males and 17 females, age: 35 ± 7 years, weight: 74 ± 20 kg, height: 169 ± 11 cm) (see the participant flow chart in Figure 1).

### 2.3 Procedures

A detailed account of the development process of the RT protocols can be found in the supplementary materials. Participants first received three short videos (3–5 min) describing the testing procedures, the RT protocol, and an overview of how to modify exercise intensity using RPE. They then participated in a pre-test session (May 2021) that included anthropometric and performance measurements and were familiarized with the exercise protocol at the exercise science laboratory at Tel-Aviv University. After signing the consent form, the examiner provided each participant with a resistance band (NT Loop, FL United States). Two resistance levels were available for the band; men received the high resistance band while women received the low resistance band. The examiner instructed participants on how to properly complete the exercises and how to use RPE for intensity regulation. Participants in both groups were also taught how to increase the resistance in all of the exercises in the protocol by modifying the band resistance or body position. Body composition measurements were then taken, followed by a warmup prior to the performance measurements. The post-test session (July 2021) was identical to the pre-test excluding the verbal instruction component. The pre- and post-test sessions lasted approximately 90 and 50 min, respectively. Data were collected at a similar time of the day for each participant (±2 h). Participants were requested to follow a specified liquid protocol to ensure reliable body composition data (see anthropometric measurements, Section 2.5.2).

### 2.4 Training Sessions

#### 2.4.1 Effort Regulation

Participants received an explanation that effort is the process of investing mental and physical resources in a task (Halperin and Emanuel, 2020). They were then told that during the sessions, they would be requested to invest a pre-specified level of subjective effort during a set, estimated with a zero to ten RPE scale. In this scale, zero corresponds to investing no resources to complete the task (no effort) and ten corresponds to investing all available resources to complete the task (maximal effort). More specifically, ten corresponds to attempting to, but not being able to produce, greater forces in the isometric exercises (e.g., isometric knee extension); attempting to, but not being able to complete, another full range of motion repetition in the dynamic exercises (e.g., push up); or attempting to, but not being able to jump any higher, in the jumping test. To ensure adequate understanding of the instructions, the explanations were repeated and practiced by the subjects throughout the pre-test session. This was done by using RPE guided warmups for each isometric measurement prior to reaching maximal efforts (i.e., complete each task at an RPE of 4, 7, 9, and 10) which encouraged a deeper understanding of using RPE during the training sessions.

#### 2.4.2 Exercise Protocol

The intervention lasted 8 weeks and included two exercise classes per week lasting ∼45 min on Mondays and Thursdays starting at 19:30 and 20:30. To avoid a time effect bias, each group completed one session a week at 19:30 and another at 20:30. The classes were delivered live and online using Zoom video communications (California, United States) by the first author—an experienced physiotherapist and personal trainer. The instructor used WhatsApp group chats (Meta, Ca, United States) to send meeting reminders on the morning of the session and a link to a password protected zoom meeting. Participants were instructed to select a viewing mode in Zoom that only presented the instructor (i.e., “speaker mode”). While the instructor provided general instructions and feedback based on participants performance, to avoid various biases, the feedback was not personal and did not include participants’ names. In both groups, the structure of the sessions was similar: 4–6 min of warm-up, 25–35 min of moderate-intensity exercises (i.e., RPE 7/10) using the resistance band and body weight exercises, and 5 min of cool-down. The RT section was composed of super-sets in which one exercise targeted the upper body and the other targeted the lower body (See Table 1 for the exercise protocol). In both groups, participants had up to 1 min to complete a set before moving on to the next set. If they completed the set in less than 1 min, they were asked to wait until the instructor guided them to the next set. This duration was selected based on our pilot work in which we found that most trainees completed the set in under 1 min in both conditions (range of 20–50 s). Certain aspects of the protocol were modified every 2 weeks (See Supplementary Material S1 for the Consensus on Exercise Reporting Template Table and video demonstration of the exercise protocol). If participants missed a live session, they were provided with written and photographed instructions on how to complete the session on their own.

**TABLE 1:** RT intervention protocol and progression. Note that participants were able to modify the exercise difficulty level by changing the tension in the resistance band and/or range of motion.

Pair #	Exercise		Weeks			
			0–2	2–4	4–6	6–8
1	Squat	Standing row	2 sets	3 sets	2 sets	2 sets
2	Dead lift	Push up	2 sets	2 sets	2 sets	2 sets
3	Banded side steps	Shoulder abduction	2 sets	3 sets	2 sets	3 sets
4	Sumo-squat	High pull			2 sets	3 sets
5	Biceps curls	Trunk rotation	2 sets	2 sets	2 sets	2 sets
	Isometric plank				2 sets of 30 s	2 sets of 45 s

The groups differed in the RPE approach used by the participants. Those in the fixed-repetition group were instructed to complete ten repetitions per set and exercise, while aiming to reach an RPE of 7/10 at the 10th repetition. Thus, participants had to select and adjust the band resistance or their body orientation, to achieve this goal. Conversely, participants in the open-repetition group were instructed to complete as many repetitions as required to reach the target RPE of 7/10 by the end of the set, using whichever resistance they preferred. Hence, participants could have selected lower resistance and completed more repetitions, selected higher resistance and completed fewer repetitions, or any other combination they preferred. Participants were also encouraged to explore different resistance and repetitions combinations between sets and exercises within and between the sessions. In both cases, participants were asked to adjust the resistance between, but not during, the sets.

### 2.5 Outcome Measures

#### 2.5.1 Adherence

The instructor documented the attendance of each participant in each session for each group. Adherence was calculated as the number of participants who attended a live session, divided by the total number of participants in each group, in each of the 16 sessions.

#### 2.5.2 Anthropometric Measurements

Anthropometric measurements included weight, height, body mass index (BMI), and fat free mass (FFM). Standing height was calculated using a SECA stadiometer. Weight and bioimpedance was measured using the SECA mBCA 515 (SECA, Hamburg, Germany), a valid and reliable analyzer of body composition (Bosy-Westphal et al., 2013). Data was stored and processed using SECA analytics 115 version 1.4.1010.6657 (SECA, Hamburg, Germany). For reliable and consistent measures, participants were requests to avoid alcoholic beverages 24 h before measurements, to avoid caffeinated products 2 h prior to the session, and to drink at least 400 ml of water up to 30 min before the test and to urinate immediately before the measurements. Participants reported liquid consumption (e.g., amount of water consumed, any deviation from protocol); this was recorded and repeated at post-test.

#### 2.5.3 Performance Measurements

Participants warmed up for 5 min with dynamic stretching and calisthenics, and then performed a specific warm up for each of the five tests. The specific warmup test was guided using RPE to regulate intensity and to allow for a deeper understanding of how to use RPE during the training intervention. Excluding the push up test, the specific warm up consisted of completing the tests with progressively higher efforts (i.e., repetitions of 2, 2, and 1 at an RPE of 4, 7, 9, respectively) prior to completing the tests with maximal effort (i.e., RPE of 10). For each test, participants completed three attempts of maximal effort, and a fourth one if their results continued to improve above 5% relative to any of the previous attempts. Each isometric contraction lasted 3 s with two-minutes of rest between attempts. The mean value of the two highest scores was analyzed. All force data were recorded using the Kforce Pro app (Kinvent, Orsay, France). For the push up tests, the maximal number of repetitions was recorded and analyzed. Prior to each trial, participants were reminded that they should perform the test with maximal effort. To maintain similar testing conditions and to avoid various biases, no verbal encouragement was provided during any of the tests. Performance measurements were performed in the following order.

### 2.5.3.1 Isometric Mid-Thigh Pull (IMTP)

The IMTP was performed with participants standing on a commercially available portable force plate (Deltas, Kinvent, Orsay, France) to record ground reaction forces at a sampling frequency of 200 Hz. Participants applied force into the ground by pulling a barbell that was secured by ratchet straps to a Smith machine (Insight Fitness, DR030B). Bar height was set to mid-thigh and was personalized by measuring hip and knee angles of 135°–150° and 125°–150°, respectively, using a goniometer. Participants were asked to hold the bar at shoulder width using an overhand grip which remained constant across the two testing sessions.

#### 2.5.3.2 Counter Movement Jump (CMJ)

CMJ was measured with the same force plate. Participants were asked to jump as high as possible with their hands on their waist. No restriction was imposed on how low they could squat before jumping. Maximal jump height (cm) was collected and determined by the vertical velocity of the center of mass at takeoff, calculated by double integrating the vertical ground reaction force through the impulse momentum method.

#### 2.5.3.3 Hand Grip Strength

Participants were seated on a stable chair without arm support. They were requested to hold the grip dynamometer (Grip, Kinvent, Orsay, France) with their dominant arm (defined as writing hand) in an extended position while their non-dominant arm was placed across their chest and legs supported on the floor.

#### 2.5.3.4 Isometric Knee Extension

Participants were seated on a large stable table without back support with the knee of their dominant leg (defined as the leg used to kick a ball) at an angle of 100–110°, as measured with a goniometer. Their shin was inserted into a padded strap which was attached to a load cell (Link, Kinvent, Orsay, France), secured to the other end of the table.

#### 2.5.3.5 Push-Up

Participants were asked if they thought they could complete horizontal push-ups on the floor. Based on their answer, participants either completed the test horizontally on the floor, or in a positive inclination that was individualized per participant by modifying the height of the Smith machine barbell on which the test was completed. Once the appropriate inclination was identified, 5–8 repetitions were completed as part of the warmup. To ensure full range of motion, a padded box (10 cm) was placed under participant’s chest, which they were requested to lightly touch with every repetition. Following a two-minute rest, participants were asked to complete as many push-ups as possible, corresponding to an RPE of ten. If the participants completed two repetitions in a row with limited range of motion, the test was stopped, and the last complete repetition performed was documented.

#### 2.5.4 Questionnaires

To gain insight on participant’s experiences with various aspects of the intervention, they were asked to answer 11 online questions every other week (Qualtrics XM Platform, Utah, United States). The questions were composed of three general satisfaction categories: 1) the exercise program, 2) the ease and clarity of using RPE as a tool to modify exercise intensity, and 3) the online setup, including the quality of the sound and video. Answers were provided using a visual analog scale (VAS) ranging from −100 (low agreement/negative) to 100 (high agreement/positive), since these scales are recommended for online platforms and are better suited to be treated as a continuous variable in statistical analyses than Likert scales (Funke and Reips, 2012; Reips and Funke, 2008). The following statements were included: 1) “Participating in the exercise program is a positive experience”. 2) “I exercised according to my preferences”. 3) “The way I exercise is aligned with my interests”. 4) “I feel that I have the opportunity to make choices with regard to the way I exercise”. 5) “Regulating workout intensity using RPE is clear to me”. 6) “I can successfully regulate exercise intensity using RPE”. 7) “I am satisfied with the variety of exercise in the program”. 8) “I find the resistance band comfortable to use”. 9) “My enjoyment levels from the last four sessions attended”. 10) “My experience with zoom—video and audio quality”. 11) “My experience with zoom—communication with the instructor”. Questions 2 to 5 were taken from the basic psychological needs in exercise scale questionnaire (Vlachopoulos and Michailidou, 2006) and were translated and back translated to Hebrew. The rest of the questions were specifically developed for this study, and were thus not validated.

### 2.6 Statistical Analysis

We tested the normality of the data *via* kurtosis and skewness inspection, in which skewness <2 and kurtosis >7 were considered as substantial deviations from normality (West et al., 1995). In cases where the normality assumption was not violated, we presented the data as mean ± standard deviation (SD). We analyzed adherence rates by fitting a repeated measure logistic regression to the longitudinal binary data, with the within-subject effect of time (16 RT sessions) and the between-subject effect of group (fixed-repetition/open-repetition). To examine if a difference occurred between groups in body weight and composition, as well as the five performance tests, we ran ANCOVAs, in which the post-test result was the dependent variable, the group was an independent variable, and the pre-test result, age and sex were used as the covariates. Additionally, we used independent *t*-tests to evaluate the change score (post-pre) differences between groups, and paired *t*-tests to evaluate the differences between pre- and post-results within each group.

To analyze the questionnaire data, we first conducted a principal component analysis on the 11 items with oblique rotation (direct oblimin) at the first time point. We used the Kaiser–Meyer–Olkin (KMO) measure to verify the sampling adequacy for the analysis (KMO = 0.66), and to confirm that all KMO values for individual items were greater than 0.5. We retained three components that explained 69% of the variance. The items that cluster on the same component suggest that component-1 represents general satisfaction from following the exercise program, component-2 represents satisfaction from using the RPE to regulate effort, and component-3 represents satisfaction from using the online platform. The average scores of items that cluster on the same component were calculated at every time point (satisfaction-program, satisfaction-RPE, satisfaction-technological). As the scores violated the normality assumption, they were compared by non-parametric tests. We compared the scores between the two groups by the Mann-Whitney U test at each of the four time points. Then, the differences between the scores measured at four time points were analyzed by Friedman’s two-way ANOVA.

Conservative multiple comparison adjustment to *p*-values was performed using the Holm-Bonferroni correction. Differences were considered statistically significant when the corresponding *p*-values were <0.05. When relevant, 95% confidence intervals (CIs) were reported. All statistical analyses were conducted using SPSS (IBM Corp. Released 2020. IBM SPSS Statistics for Windows, Version 28.0. New York, United States).

## 3 Results

No adverse events were recorded or reported during the intervention. Eleven participants dropped out from the study (four from the fixed-repetition and seven from the open-repetition group). Three participants dropped out due to health issues unrelated to the study, and eight participants dropped out due to time constraints. All the raw data is provided in the Supplementary Material S2 file.

### 3.1 Adherence Rates

**FIGURE 2:**
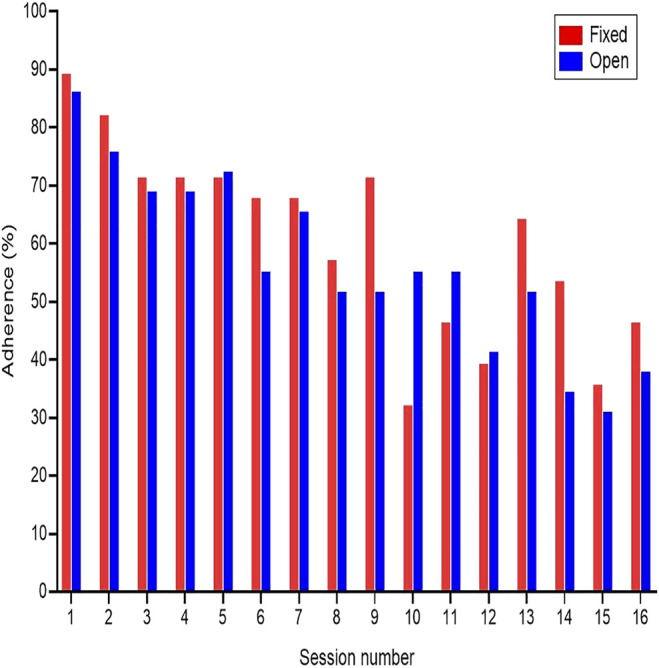
Adherence rates.

When including all participants in the analysis, the average adherence rates across sessions was 60% in the fixed-repetition and 56% in the open-repetition groups. We did not identify a statistically significant differences between the two groups $\chi^{2}$ [ $\chi^{2}$ (1) = 0.40, *p* = 0.525], nor an interaction between groups and time $\chi^{2}$ [ $\chi^{2}$ (15) = 18.93, *p* = 0.217]. However, a statistically significant effect of time was observed $\chi^{2}$ [ $\chi^{2}$ (15) = 133.53, *p* < 0.001] in which adherence rates across groups decreased from 89% at the first session, to 42% by the 16th session (Figure 2).

When excluding from the analysis participants who officially dropped out within the first three sessions (two participants from fixed-repetition and one from open-repetition), and those who did not attend a single session (one participants from fixed-repetition), the average adherence rates across sessions was 64% in the fixed-repetition and 60% in the open-repetition group. We did not identify a statistically significant difference between the two groups $\chi^{2}$ [ $\chi^{2}$ (1) = 0.64, *p* = 0.426], nor an interaction between groups and time $\chi^{2}$ [ $\chi^{2}$ (15) = 22.08, *p* = 0.106]. However, a statistically significant effect of time was observed $\chi^{2}$ [ $\chi^{2}$ (15) = 168.76, *p* < 0.001] in which adherence rates decreased from approximately 90% at the first session, to 45% by the 16th session.

### 3.2 Body Weight, Composition and Performance Test

While no significant effect of group was observed for any of the variables, some improvements from pre- to post-tests were observed. Mainly, participants increased their fat-free mass by 0.3 kg (95% CI: 0.1–0.6), isometric mid-thigh pull by 5.5 kg (95% CI: 0.8–10.4), isometric knee-extension by 2.2 kg (95% CI: 0.8–3.7), and push-ups by 6.3 repetitions (95% CI: 4.5–8.2) (Tables 2, 3).

**TABLE 2:** Descriptive statistics of the pre- and post-intervention results in body composition and performance outcomes in each group and across groups.

	Fixed-repetition (*n* = 24)		Open-repetition (*n* = 22)		Both (*n* = 46)	
	Baseline Mean ± SD	8 weeks Mean ± SD	Baseline Mean ± SD	8 weeks Mean ± SD	Baseline Mean ± SD	8 weeks Mean ± SD
Body weight (kg)	73.4 ± 17.1	73.0 ± 17.6	71.1 ± 18.6	71.2 ± 18.2	72.3 ± 17.7	72.1 ± 17.7
Fat free mass (kg)	47.1 ± 10.8	47.5 ± 10.7	50.8 ± 14.0	51.1 ± 13.3	48.9 ± 12.4	49.2 ± 12.0
BMI (kg/m^2^)	25.8 ± 5.6	25.6 ± 5.7	24.6 ± 4.4	24.7 ± 4.2	25.2 ± 5.05	25.1 ± 5.03
IMTP (kg)	183.6 ± 40.6	189.8 ± 48.2	197.2 ± 63.3	201.9 ± 60.0	190.1 ± 52.6	195.6 ± 53.9
Jump height (cm)	20.6 ± 5.4	21.0 ± 5.2	24.4 ± 7.2	24.9 ± 7.5	22.3 ± 6.5	22.8 ± 6.6
Grip force (kg)	27.3 ± 7.6	27.0 ± 8.02	28.3 ± 9.4	29.1 ± 9.7	27.8 ± 8.5	28.0 ± 8.8
MVC knee (kg)	35.6 ± 11.5	37.9 ± 10.4	40.7 ± 16.4	42.8 ± 17.7	38.0 ± 14.1	40.3 ± 14.4
Pushups (reps)	17.7 ± 9.6	24.5 ± 11.9	17.7 ± 6.9	23.6 ± 8.8	17.7 ± 8.3	24.1 ± 10.4

**TABLE 3:** Inferential statistics for the body composition and performance tests between groups (ANCOVA and unpaired *t*-tests on change scores) and within groups (paired *t*-tests on pre- and post-test results) analysis. *p* values, point estimate and 95% CI are reported.

	Between group differences		Within (post-pre) group differences		
	Fixed–Open (ANCOVA)^a^	Fixed–Open (unpaired *t*-test)	Fixed-repetition (*n* = 24)	Open-repetition (*n* = 22)	Both (*n* = 46)
Body weight (kg)	*p* = 0.57	*p* = 0.38	*p* = 0.24	*p* = 0.95	*p* = 0.40
	0.30 (−0.77, 1.38)	−0.44 (−1.47, 0.57)	−0.43 (−1.16, 0.31)	0.02 (−0.72, 0.77)	−0.21 (−0.72, 0.29)
Fat free mass (kg)	*p* = 0.84	*p* = 0.83	*p* = 0.04	*p* = 0.30	*p* = 0.04
	0.06 (−0.53, 0.64)	0.06 (−0.53, 0.65)	0.33 (0.01, 0.65)	0.27 (−0.26, 0.80)	0.31 (0.01, 0.59)
BMI (kg/m^2^)	*p* = 0.34	*p* = 0.18	*p* = 0.14	*p* = 0.70	*p* = 0.41
	0.18 (−0.20, 0.57)	−0.24 (−0.62, 0.12)	−0.20 (−0.46, 0.07)	0.05 (−0.22, 0.32)	−0.08 (−0.26, 0.10)
IMTP (kg)	*p* = 0.93	*p* = 0.76	*p* = 0.09	*p* = 0.15	*p* = 0.02
	0.41 (−9.18, 10.00)	1.45 (−8.18, 11.1)	6.23 (−1.07, 13.52)	4.77 (−1.85, 11.39)	5.53 (0.76, 10.39)
Jump height (cm)	*p* = 0.61	*p* = 0.84	*p* = 0.97	*p* = 0.37	*p* = 0.15
	0.35 (−1.05, 1.74)	0.12 (−1.41, 1.74)	0.39 (−0.26, 1.06)	0.53 (−0.68, 1.73)	0.45 (−0.17, 1.09)
Grip force (kg)	*p* = 0.35	*p* = 0.19	*p* = 0.62	*p* = 0.16	*p* = 0.60
	0.75 (−0.84, 2.36)	−1.03 (−2.58, 0.52)	−0.28 (−1.45, 0.89)	0.74 (−0.32, 1.82)	0.20 (−0.57, 0.99)
MVC knee (kg)	*p* = 0.98	*p* = 0.91	*p* = 0.04	*p* = 0.04	*p* = 0.003^b^
	0.04 (−3.01, 3.17)	0.16 (−2.72, 3.1)	2.29 (0.14, 4.43)	2.12 (0.08, 4.15)	2.21 (0.78, 3.64)
Pushups (reps)	*p* = 0.55	*p* = 0.60	*p* < 0.001^b^	*p* < 0.001^b^	*p* < 0.001^b^
	−1.13 (−4.97, 2.71)	0.97 (−2.72, 4.66)	6.83 (4.06, 9.6)	5.86 (3.28, 8.44)	6.36 (4.53, 8.20)

a The coefficient of group variable in the ANCOVA model where the fixed-RPE group was used as a reference.

b Statistically significant results at the significance level of 5% according to Holm-Bonferroni method.

### 3.3 Questionnaires

**FIGURE 3:**
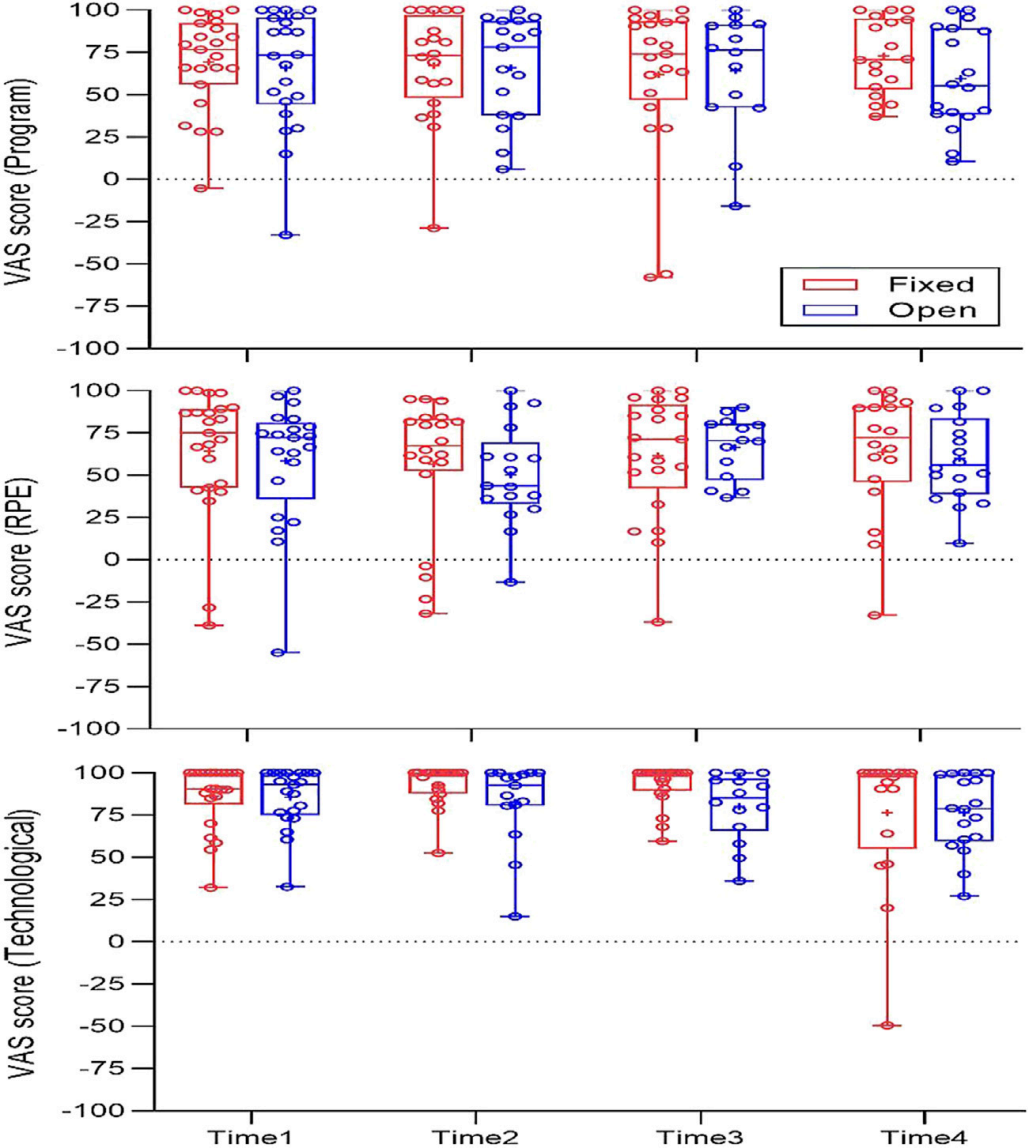
Questionnaire results.

Participants that attended less than three sessions (*n* = 10) were excluded from this analysis as the questionnaires concerned the evaluation of the program on an ongoing basis (e.g., “Your enjoyment from the last four sessions attended”). Excluding one significant difference between groups in the technological satisfaction factor at time-3, favoring the fixed-repetition approach, no significant differences were observed between groups across time points using the Mann-Whitney U test (Figure 3). We analyzed the aggregated data of the two groups (each time point included 35–45 responses per component), and compared the calculated scores between the four time points using Friedman’s two-way ANOVA. Excluding one significant difference in the RPE factor between time-2 and time-1, we observed no significant differences between the scores within each factor across time points (Figure 3). The median satisfaction rates in all components, across groups and time points, ranged between 44 and 100 in the −100 to 100 VAS scale.

## 4 Discussion

We compared two RPE based approaches to prescribe RT intensity during live, online, group RT sessions, over a period of 8 weeks, among participants with no experience in RT. Whereas the results of all outcomes were similar between the fixed and open-repetition approaches, participants in both groups increased their fat free mass, improved performance in some outcome measures, and enjoyed exercising according to the protocol. Coupled with the zero reports of adverse events, our study is aligned with others (Mascarenhas et al., 2018; Schwartz et al., 2021b; Kikuchi et al., 2021), showing that videoconferencing is a safe, effective, and a cost-effective method to deliver exercise sessions. However, adherence rates gradually decreased during the study, which suggest that some amendments to the intervention may be required.

While the lack of differences between groups in all outcomes may stem from the small sample size and the relatively short duration of the intervention, the overall results have practical implications. Mainly, implementation of a specific RPE prescription approach can be based on the trainers or trainees’ preferences. Indeed, it is reasonable to expect that matching the RT prescription approach to one’s preferences may positively impact enjoyment and adherence rates (Teixeira et al., 2012; Rodrigues et al., 2020). To illustrate, in two recent studies from our laboratory, participants completed RT protocols in which the number of repetitions was either fixed, based on one’s ongoing RPE (Schwartz et al., 2021a) or self-selected out of prescribed range (Emanuel et al., 2021). After completing both RT sessions, participants reported which of the two approaches they preferred. In both studies, approximately half of the participants preferred the fixed repetition approach while emphasizing the importance of having a clear set endpoint. Conversely, the other half of participants preferred the RPE based and the self-selected approaches, while emphasizing the importance of having control over when to terminate a set based on their ongoing perceptions. While in the current study participants were randomized into one of the two groups, future research could inspect the effects of allowing participants to exercise according to their preferred approach. It is possible that doing so will lead to higher adherence rates.

Across the two groups, adherence levels began at 89% and gradually decreased to 42%, a result that is lower than the rates reported in similar studies (Schwartz et al., 2021b; Ibrahim et al., 2021). The relatively low adherence rates are not consistent with the fact that both groups rated the program as enjoyable and that it elicited high perceptions of autonomy, both of which are associated with higher adherence rates (Gjestvang et al., 2021; Teixeira et al., 2012). This inconsistency can be partly explained by the hours in which the sessions took place, specifically the 19:30 to 20:15 session, which was reported as a key reason for missed sessions, mostly by participants with young children. In addition, to avoid various biases, the verbal feedback provided by the instructor excluded personal feedback. To illustrate, in case the instructor spotted a participant completing an exercise with faulty technique, she provided a general feedback statement to all participants regarding how the exercise should be performed without mentioning the participant’s name. Participants were also instructed to select “speaker mode” in Zoom, meaning that they were only able view the instructor during the sessions. The absence of personal feedback and the inability to view and relate to the other group members may have also negatively impacted adherence rates. Future research aiming to implement similar designs and increase adherence rates should consider these points when planning the study.

Participants in both groups improved their performance in the isometric mid-thigh pull, isometric knee extension, and push-ups test, but not in the gripper and countermovement jump. These results are directionally aligned with studies that implemented similar designs (Orange et al., 2020; Aksay, 2021; Kikuchi et al., 2021). The performance improvements could have been limited by several reasons. First, we analyzed the results of all participants who completed the post-tests, irrespective of how many sessions they attended. In view of the low adherence rates, the performance improvement may have been attenuated in those who did not comply with the program. Second, given the large number of post-tests (*n* = ∼25) that had to be conducted shortly after the last session of each group, some participants were tested 7 days after their last trainings session. This delay, which was comparable between groups, could have negatively affected the performance results. Third, we selected time efficient, easy to administer performance tests that had a short learning curve. However, excluding the push-up test, the rest of the performance tests did not fully resample the exercises performed in the intervention. Since improvements in performance are larger when the tests match the practiced exercises (Rutherford and Jones, 1986; Morrissey et al., 1995), the limited performance improvement can be partly explained by the implemented tests.

Several methodological concerns of this study are worthy of discussion. First, we did not conduct a power analysis to determine the sample size. Thus, the lack of differences between groups in all outcomes may stem from a type two error. Second, the examiners who collected the pre- and post-tests data were aware of which group the participants they tested belonged to. While the examiners strictly followed scripted guidelines and a standardized testing protocol, the lack of blinding could have introduced some biases. Third, the results of this study are limited to healthy and young participants without any RT experience. The overall intensity of the implemented intervention in this study may not be enough to elicit meaningful adaptations among participants with RT experience. For such cohorts, modification to the intervention may be required.

## 5 Conclusion

We observed non-significant and trivial differences between groups in all outcomes. Participants in both groups increased their fat free mass, improved their performance in most, but not all tests, and reported high satisfaction rates with the program. However, in both groups, adherence rates gradually declined during the study. Some aspects of the protocol may require modifications in the future, such as reconsidering the time of the day of the classes and the type of feedback provided, in order to improve adherence rates.

## Data Availability

The original contributions presented in the study are included in the article/Supplementary Material, further inquiries can be directed to the corresponding author.

## Footnotes

1 While RT is commonly associated with the implementation of barbells and dumbbells, RT can also be performed with one’s bodyweight, resistance bands, and more. Hence, in this article we use the term resistance to include all possibilities that can be implemented in RT.
