# Supplementary material for: Prescribing Intensity in Resistance Training Using Rating of Perceived Effort: A Randomized Controlled Trial
Source: Front Physiol. 2022 Apr 29;13:891385. doi: 10.3389/fphys.2022.891385 (PMC9099373; doi:10.3389/fphys.2022.891385)
Supplement: Supplementary file 1 [file Table1.docx]

Supplementary materials for “Delivering [web-based resistance training group sessions using rating of perceived effort: a randomized](https://journals.sagepub.com/doi/full/10.1177/1357633X18768400) controlled trial”

[Design of the intervention 2](#_Toc89783072)

[Consensus on Exercise Reporting Template (CERT) 4](#_Toc89783073)

# Design of the intervention

*Protocol*

Developing the two protocols required approximately 10, one-hour team meetings, and 30 hours of pilot sessions. The research team included two physiotherapists (Y.BZ and H.S) and two personal trainers (I.HN and I.H) with extensive experience in training a wide range of populations. During the team meetings, we attempted to integrate the best available evidence from established RT guidelines, while considering foreseeable logistical challenges and ways to adjust exercise intensity in an individualized and time efficient manner. We agreed that we might modify some aspects of the protocol after a few weeks in view of the feedback from surveys collected every other week.

*Equipment*

We decided on the NT-Loop band based on our own experience, and because it consists of a single band that can be used across different exercises and allows comfortable and substantial modification of the resistance. This is in contrast to other types of bands which work well with some exercises but not others, and may thus require a range of different bands to complete effective RT sessions.

*Pilot*We first developed the explanation narrative for the fixed-repetitions RPE group, and piloted single sessions on five adults during which the time to complete each set per exercise was documented. After each pilot session, we asked the participants to share their experiences (e.g., perceived level of difficulty and the ease in which they were able to understand and use RPE in adjusting the intensity). We learned that the time required to complete ten repetitions varied between participants and exercises, and took between 30 and 50 seconds. The same process was then repeated for the open-repetitions RPE instructions. The protocol was delivered individually to five adults and the researcher documented their experiences and the time trainees took to complete each set. Given the greater variability in number of repetitions trainees completed, we found that one minute was the upper limit trainees took to complete a set. Hence, we decided that one minute would be the time dedicated to a given set in both groups before moving to the next exercise. Once the duration of sets was established, each protocol was delivered online to a group of 2-3 participants on three different occasions (total of six sessions). Trainees were instructed to wait once they completed their set until the instructor provided the cue to start next the exercise.

*Effort regulation* Participants received an explanation that effort is the process of investing mental and physical resources in a task. This explanation prior to pre-test and explained further explained and demonstrated during the session. For example, participants were guided to modify resistance for the biceps curl exercise. The closed repetition group rated RPE after ten repetitions and were then asked to modify the resistance to reach PE 7 at the 10^th^ repetition. The open repetition group also performed the exercise twice but was first ask to use light resistance and complete as many repetitions needed to reach RPE 7, the same was done for heavier resistance.

*Motivation*

In order to promote motivation and adherence to exercise, the instructor emphasized positive and encouraging feedback throughout the sessions (e.g., “Good job, you’re doing great”), which is known to increase positive affect, the sense of competence, and future intentions to exercise. Participants were encouraged to contact the instructor for any questions regarding exercise modification or any aspect of the protocol. This was possible following each session during which the instructor stayed online for 10 more minutes after the session ended, or via WhatsApp group chats that were also used by the instructor to deliver reminders, attendance encouragement, and distribute questionnaires every other week. To control for confounders and reduce risk of biases, we decided against providing personal feedback during the training sessions. It should be noted that the lack of individual personal attention may negatively impact adherence, and should be considered when implementing this protocol in the field.

*Technology*

We selected the Zoom video-conferencing application due to its popularity, user-friendly interface, and our experience with it conducting a similar study. An additional benefit is that Zoom does not require participants to pre-install it on their devices nor does it require preregistration. Zoom allows different screen layouts such as spotlight view meaning the person marked is presented on full screen versus gallery view in which all meeting participants are presented on the screen. To control for confounders and increase consistency, the instructor was spotlighted, and participants were instructed to constantly use the same device, location, and settings throughout the intervention.

| Consensus on Exercise Reporting Template (CERT) | | |
| --- | --- | --- |
| Item | Description of guideline | Implementation |
| 1 | Detailed description of the type of exercise equipment | The intervention consisted of online resistance training sessions, delivered live, twice a week, with each session lasting ~45-minutes. The exercise equipment included a resistance band provided by the research team (<https://www.ntloop.com/shop/>), a stable chair, and an exercise mat. |
| 2 | Detailed description of the qualifications, expertise and/or  training | The exercise intervention was designed by two certified strength and conditioning Specialists and two physiotherapists and was delivered by the first author who is a physiotherapist, personal trainer, and Pilates instructor. |
| 3 | Describe whether exercises are performed individually or in a group | The exercise sessions were delivered by a single instructor to a group of participants exercising at their homes, via video-conferencing interface "Zoom video communications" (California, USA). Participants were instructed to mute their microphones during the sessions, and to view the session in speaker view. This means the participants had no visual interaction with other participants during the sessions. |
| 4 | Describe whether exercises are supervised or unsupervised; how they are delivered | The instructor remotely supervised the training sessions. The instructor verbally guided participants on how to perform the exercises and how to regulate exercise intensity. However, feedback was only delivered at the group level. Individual feedback was available after the class during which the instructor stayed online for 5-10 minutes in case that specific questions arose. |
| 5 | Detailed description of how adherence to exercise is measured and reported | Attendance was tracked and saved in a logbook throughout the intervention. Additionally, all sessions were recorded and saved to the cloud for future inspection. Early leave or late entry of up to 20 minutes was documented as attendance. Adherence was calculated as: [number of participants who attended a session in a group divided by the number of total participants in the group] x 100. This was done for all 16 sessions. |
| 6 | Detailed description of motivation strategies | the instructor provided positive and encouraging feedback throughout the sessions. We also created a WhatsApp groups for each group in which reminders were sent in the morning of the workout and 15 minutes before the session started, and reinforcements for participating the following day. |
| 7a | Detailed description of the decision rule(s) for determining exercise progression | The exercise protocol was modified twice a month as a function of survey responses, the professional assessment of the instructor, and staff discussions. Throughout the program we made slight modifications to the basic protocol regarding exercise selection, order, and number of sets, leading to four phases for the exercise protocol. |
| 7b | Detailed description of how the exercise program was progressed | The instructor routinely explained how to regulate intensity for each exercise by modifying the resistance band length, range of motion, and body orientation. Phase-1 included four super-sets that were repeated twice each, leading to 16 sets per session. In phase-2, we replaced the hip thrust exercise with banded side steps, and added another set of the following supersets: squat/row and banded side steps/shoulder abduction, which increased the total number of sets to 20 per session. In phase-3 we added a new super-set (sumo squat /high pull) that was performed twice and two sets of 30 seconds plank at the end of the RT section, which increased the total number of sets to 24 per session. In phase-4 we added 4 sets by repeating following supersets: sumo squat/high pull, banded side steps/shoulder abduction, leading to a total of 28 sets per session. |
| 8 | Detailed description of each exercise to enable replication | Exercise protocol for each phase is available at:  <https://youtube.com/playlist?list=PL4BdVVEc6Cb018TBa9vyl_xCTy8QA6T38> |
| 9 | Detailed description of any home program component | Participants were asked to maintain their usual physical activity levels and not to change their nutritional habits. |
| 10 | Describe whether there are any non-exercise components | This intervention was solely exercise based. |
| 11 | Describe the type and number of adverse events that occur during exercise | None. |
| 12 | Describe the setting in which the exercises are performed | Participants completed the training intervention in their home environment. They had to have a stable internet connection and a camera located in a place that allowed the instructor to observe the participant exercising. |
| 13 | Detailed description of the exercise intervention | Each session included a five-minute warm-up, 30-minutes of RT, and a five-minute cool down. The RT section was composed of super-sets in which one exercise targeted the upper body and the other targeted the lower body. Super-sets were performed sequentially with a rest between super-sets. |
| 14a | Describe whether the exercises are generic (one size fits all) or tailored | The exercise intensity was tailored in the sense that each participant was able to modify the resistance of each exercise to reach a RPE of 7/10 by the end of each set. |
| 14b | Detailed description of how exercises are tailored to the individual | Following the online and in person explanation of how to modify exercise intensity using RPE, participants had to demonstrate each exercise with sufficient technique. During this session, slight modifications and individualization of the exercise were made to suit the participant (e.g., modify standing width, range of motion). |
| 15 | Describe the decision rule for determining the starting level | The number of exercises, sets, and rest period seemed suitable based on the shared experience of the research team, on pilot work, similar research projects, and relevant literature. |
| 16a | Describe how adherence or fidelity is assessed | Adherence was calculated as the number of participants who attended a session, divided by the total number participants in each group. This was done for each of the 16 total sessions. This proportion was presented and analyzed as percentages. |
| 16b | Describe the extent to which the intervention was delivered as planned | Other than a few expected modifications, the intervention was delivered as planned. |
